# Prevalence of Dental Caries and Fissure Sealants in a Portuguese Sample of Adolescents

**DOI:** 10.1371/journal.pone.0121299

**Published:** 2015-03-24

**Authors:** Nélio J. Veiga, Carlos M. Pereira, Paula C. Ferreira, Ilidio J. Correia

**Affiliations:** 1 Health Sciences Research Centre – Health Sciences Faculty, Beira Interior University, Covilhã, Portugal; 2 Research Centre for Education, Technology and Health Studies – Polytechnic Institute of Viseu, Viseu, Portugal; 3 Chemical Process Engineering and Forest Products Research Centre, Chemical Engineering Department, University of Coimbra, Coimbra, Portugal; 4 Health Sciences Department – Portuguese Catholic University, Viseu, Portugal; Taipei Medical University, TAIWAN

## Abstract

**Introduction:**

The aims of this study were to assess the prevalence of dental caries and the DMFT index, as well as the distribution pattern of pit and fissure sealants on permanent teeth in a Portuguese sample of adolescents, and to assess whether the existing usage of sealants and socio-demographic factors are correlated to caries prevalence on the examined sample.

**Materials and Methods:**

A cross-sectional study was designed with a sample of 447 adolescents aged 12 to 18 years old, attending a public school in Sátão, Portugal. A self-administered questionnaire with questions about oral health behaviours and socio-economic status was answered by adolescents in the classroom. Clinical examination of oral health status and assessment of fissure sealants were accomplished by only one trained member of the research team.

**Results:**

We obtained a DMFT index of 3.32 (2.92), which indicates a moderate level of prevalence of dental caries. When considering a DMFT = 0, we found significant statistical differences between the parents´ level of education (≤ 4^th^ grade = 26.3 vs 5^th^–12^th^ grade = 18.8 vs <12^th^ grade = 43.3, p = 0.001), gender (male = 27.3 vs female = 19.6, p = 0.04), age (≤15 years = 27.1 vs <15 years = 18.5, p = 0.02), presence of fissure sealants (yes = 30.6 vs no = 13.5, p = 0.001) and experience of dental pain (no = 25.4 vs yes = 16.8, p = 0.02). When analyzing the prevalence of fissure sealants, we verified that 58.8% of adolescents had at least one fissure sealant applied. Significant statistical differences were found when analyzing the presence of fissure sealants related with parents´educational level (<9^th^ grade, OR = 1.56 CI95% = 1.05–2.54), gender (female, OR = 1.86 CI95% = 1.19–2.98), experience of dental pain (yes, OR = 0.62 CI95% = 0.39–0.97) and presence of dental caries (yes, OR = 0.35 CI95% = 0.19–0.65).

**Conclusions:**

The moderate level of caries prevalence reveals the need of improvement of primary prevention interventions among Portuguese adolescents. The establishment of a more targeted preventive program with better and more effective oral health education is essential, having into account socio-demographic aspects.

## Introduction

In the last decades the World Health Organization (WHO) has dedicated special attention to oral health and the prevalence and causes of oral diseases, namely dental caries and periodontal diseases, and, most recently, oral cancer.[1]

The risk of development of oral diseases, namely dental caries and periodontal diseases, is strongly related with lifestyle habits. Health-promoting lifestyles include infrequent sugar consumption, toothbrushing effectively and regularly at least twice a day, daily use of dental floss and visiting a dentist regularly to prevent and detect oral diseases in an early stage. Most researchers also believe that oral health knowledge and parents’ attitudes mostly determine and promote children’s oral health behaviour.[1–3]

One of the most important oral health indicators used in epidemiological studies is the decayed, missing and filled permanent teeth index (DMFT index) developed by Klein and Palmer in 1937.[4,5]

After obtaining the DMFT index, it is possible to determine the level of severity of dental caries in the study sample: very low (DMFT index between 0.1 and 1.1); low (DMFT index between 1.2 and 2.6); moderate (DMFT index between 2.7 and 4.4); high (DMFT index between 4.5 and 6.5) and very high (DMFT index higher than 6.5).[1]

In Portugal, dental healthcare is mainly provided by the private sector. In the past years, several oral health programs have been developed in order to finance the dental treatments of the poorer socioeconomic status to compensate the very low number of dental appointments in the public sector (health centers and hospitals). The oral health programs are mainly addressed to children and adolescents, focusing mainly on primary prevention with the application of fluoride products and fissure sealants in the dental appointment.[6,7]

Pits and fissures on occlusal surfaces of permanent teeth are particularly susceptible to the development of dental caries. This susceptibility to dental caries is related with the individual morphology of the tooth´s pits and fissures, which can be prosperous shelters for microorganisms and make the hygiene procedures of these areas more difficult, allowing greater plaque aggregation.[8] Consequently, with a view to preventing dental caries, fissure sealant application is recommended if pits and fissures are very deep and narrow, thus creating a physical barrier for the plaque's accumulation in these specific anatomical areas of the tooth.[9] This primary prevention measure is highly recommended to prevent dental caries formation, but also to control and arrest the development of incipient dental caries.[10,11] Fissure sealants are used mostly as a primary preventive measure in dental medicine.[12]

Fissure sealants are considered to be an effective and economical method for dental caries primary prevention, so it has been integrated in oral health community programs.[10,13–15] However, a study developed by Martin *et al*. (2013) concludes that dentists may be underusing fissure sealants, even when knowing that a fissure sealant is effective and has positive effects on the prevention of oral diseases.[16]

The aim of this study was to assess the prevalence of dental caries and the DMFT index, as well as the distribution pattern of pit and fissure sealants on permanent teeth (molars and pre-molars) in a Portuguese sample of adolescents, and to assess whether the existing usage of sealants and sociodemographic factors are correlated to caries prevalence on the examined sample.

## Materials and Methods

A non-probabilistic convenience sampling of 447 adolescents aged between 12 and 18 years old, attending a public school in Sátão, Portugal, was enrolled in this epidemiological observational cross-sectional study that was carried out from September to December of 2012. The studied sample represents the entire school group of the area, and 88.6% of its pupils were enrolled in the study. Questionnaires without information about gender and age were excluded from the study as well as the adolescents whose parents did not sign the informed consent before data collection.

A self-administered questionnaire with questions about sociodemographic variables, social and daily habits and oral health behaviours was filled out by adolescents in the classroom. Sociodemographic variables such as gender (male/female), age, school grade at the moment of the study, residence area (living in a village near the town of Sátão was considered rural area and living in the town of Sátão was considered urban area), parents´ educational level (choosing the higher educational level between father and mother) and father´s professional situation (employed/unemployed) were determined. Oral health behaviours were assessed by questioning adolescents about their ideas on their own oral health, the frequency of toothbrushing per day, time spent toothbrushing (in minutes), period of the day that each adolescent toothbrushed, daily use of dental floss, having a dental appointment in the last 12 months and the reason of the last dental appointment, if he or she had experienced at least one episode of dental pain during their lives, if adolescents were afraid of going to the dentist, if they knew what was a fissure sealant and if their dentist had ever applied fissure sealants during a dental appointment and finally if they frequently consumed sugary beverages or soft drinks.

Clinical examination of oral health status was carried out according to the WHO criteria to determine the prevalence of dental caries and the DMFT index.[17] Teeth were clinically examined with dental instruments by using visual-tactile method with the use of a dental mirror and a probe for cavitation detection only (approved by the WHO for caries diagnosis) and took place in the classroom under standardized conditions recommended by the WHO.[17] Cotton rolls and gauze were available to remove moisture and plaque when necessary. Data collection of the adolescent´s oral health status was registered by only one trained calibrated dentist/researcher.

The recorded variables of the clinical examination were caries experience, using the DMFT as oral health indicator, which consists in the sum of teeth decayed, teeth missing due to dental caries and teeth filled for each analyzed adolescent. The presence of fissure sealants was also assessed by clinical examination and, if present, each tooth was classified considering the following classification:

1. Completely intact fissure sealant;
2. Infiltrated fissure sealant without dental caries;
3. Infiltrated fissure sealant with dental caries;
4. Partial fissure sealant, without dental caries;
5. Partial fissure sealant, with dental caries.

Data analysis was carried out using the *Statistical Package for Social Sciences* (SPSS 18.0 version). Prevalence was expressed in proportions and crude odds ratio (OR) with 95% confidence intervals (CI) was used to measure the strength of association between variables. Proportions were compared by the Chi-square test and continuous variables by the Kruskal-Wallis and Mann-Whitney tests. The significance level established the inferential statistics was 5% (p<0.05). A multivariate analysis—logistic regression—was applied for analysis of the association between variables.

This research involving human data has been performed in accordance with the Declaration of Helsinki and was submitted and approved by the Ethics Committee of the Research Centre for Education, Technology and Health Studies of the Polytechnic Institute of Viseu, Portugal (CI&DETS).

The information collected by the questionnaires was provided voluntarily and confidentially. The anonymity of the information collected was guaranteed by telling adolescents not to sign their names or write down any other form of identification in any part of the questionnaire. Data collection taken into account only considered the answers given by adolescents whose parents signed an informed consent that explained the objectives of the study. After collection, questionnaires were numbered, stored and processed by computer. The results do not make reference to adolescents’ names or contain any information that may identify any of the participants.

## Results

The final sample was composed by 447 adolescents, 38.3% male and 61.7% female, all between the age of 12 and 18 years old, from a public school of Sátão, Portugal. When analyzing the parents’ educational level, we can verify that 4.3% have parents that only attended school up to the 4^th^ grade of the primary school, 53.5% attended school from the 5^th^ to the 12^th^ grade and 15.0% had access to a higher education degree after finishing the 12^th^ grade. The analysis of the distribution of the sample by residence area indicates that the majority live in rural areas (65.3% vs 34.7%) (Table 1).

**Table 1:** Sample characterization.

	Male		Female		Total	
	N	%	N	%	N	%
	171	38.3	276	61.7	447	100.0
Age						
12	31	18.2	27	9.8	58	13.0
13	29	17.0	38	13.8	67	15.0
14	18	10.5	34	12.3	52	11.6
15	28	16.4	27	9.8	55	12.3
16	16	9.4	34	12.3	50	112
17	16	9.4	29	10.5	45	10.1
18	33	12.3	87	24.3	120	26.6
Grade						
7	34	19.9	26	9.4	59	13.4
8	26	15.2	38	13.8	64	14.3
9	19	11.1	33	12.0	52	11.6
10	30	17.5	38	13.8	68	15.2
11	19	11.1	32	11.6	51	11.4
12	44	25.2	109	39.4	153	34.1
Parents´educational level						
≤ 4 grade	3	2.0	16	6.9	19	4.3
5–12 grade	105	61.4	134	48.6	239	53.5
> 12 grade	30	17.5	37	13.4	67	15.0
Without information	33	19.1	89	31.1	122	27.2
Residential area						
Rural	100	58.5	192	69.6	292	65.3
Urban	71	41.5	84	30.4	155	34.7

The DMFT index obtained in this study was 3.32 (2.92). We obtained a mean of 0.99 (1.72) for the decayed component of the index, 0.10 (0.40) for the missing component and 2.23 (2.37) for the filled component.

Out of the total sample, we verified by intra-oral examination that 61.0% of the adolescents did not present any dental caries at the moment of the observation, 92.6% did not lose any tooth due to dental caries and 66.5% had at least one filled tooth due to dental caries. Only 22.6% presented a DMFT = 0, which indicates the prevalence of adolescents who never had dental caries.

When analysing the DMFT by participant, we verified that 22.6% present a DMFT = 0, 34.2% DMFT between 1 and 3 and 43.2% higher than 4.

This study showed that females had a higher DMFT than males. But a lower prevalence of dental caries at the moment of data collection as 59.9% indicated that female adolescents were free of dental caries, while for the male fraction of the sample this value was 40.1%.

When associating the presence of dental caries according to the sample and sociodemographic variables we can verify that the prevalence of dental caries was lower among adolescents whose parents had a higher level of education (>9^th^ grade, OR = 0.42 CI95% = 0.25–0.72) and among those who live in an urban area (urban, OR = 0.69 CI95% = 0.41–1.15), and higher among adolescents whose father was unemployed (unemployed, OR = 1.16 CI95% = 0.51–2.65), female gender (female, OR = 1.54 CI95% = 0.97–2.45) and older (>15 years, OR = 1.87 CI95% = 1.06–3.29). When considering a DMFT = 0, we found significant statistical differences between parents´ level of education (≤ 4^th^ grade = 26.3 vs 5^th^-12^th^ grade = 18.8 vs >12^th^ grade = 43.3, p = 0.001), gender (male = 27.3 vs female = 19.6, p = 0.04) and age (≤15 years = 27.1 vs >15 years = 18.5, p = 0.02) (Table 2).

**Table 2:** Prevalence of DMFT (per individual) and association with sociodemographic variables.

	DMFT = 0		DMFT = 1 to 3		DMFT ≥4		
	N	%	N	%	N	%	*p*
**Parents´educational level**							
≤ 4^th^ grade	5	26.3	3	15.8	11	57.9	0.001
5–12 grade	45	18.8	89	37.2	105	43.9	
>12 grade	29	43.3	21	31.3	17	25.4	
Total	79	24.3	113	34.8	133	40.9	
**Father´s professional situation**							
Employed	70	23.6	102	34.5	124	41.9	0.5
Unemployed	8	21.1	17	44.7	13	34.2	
Total	78	23.3	119	35.6	137	41.1	
**Gender**							
Male	44	27.3	52	32.3	65	40.4	0.04
Female	51	19.6	92	35.4	117	45.0	
Total	95	41.3	144	32.7	182	26.0	
**Age**							
≤ 15 years	62	27.1	88	38.4	79	34.5	0.02
> 15 years	32	18.5	51	29.5	90	52.0	
Total	94	23.4	139	34.6	169	42.0	
**Residence area**							
Rural	56	19.9	97	34.5	128	45.6	0.1
Urban	39	27.9	47	33.6	54	38.6	
Total	95	22.6	144	34.2	182	43.2	

When associating the presence of dental caries and oral health behaviours, dental pain and fissure sealant application, it is possible to verify that the prevalence of dental caries was lower among adolescents who toothbrushed more frequently (≥ twice a day, OR = 0.89 CI95% = 0.46–1.71) and those to whom fissure sealants had been applied (yes, OR = 0.35 CI95% = 0.19–0.65). The prevalence of dental caries was higher among adolescents who had experienced at least one episode of toothache during their lives (yes, OR = 1.68 CI95% = 0.98–2.84). When considering a DMFT = 0, we found significant statistical differences between adolescents with and without fissure sealants applied to their teeth during intraoral examination (yes = 30.6 vs no = 13.5, p = 0.001) and when associated with the variable experience of dental pain (no = 25.4 vs yes = 16.8, p = 0.02) (Table 3).

**Table 3:** Prevalence of DMFT (per individual) and association with oral health behaviours, experience of dental pain and fissure sealant application.

	DMFT = 0		DMFT = 1 to 3		DMFT ≥4		
	N	%	N	%	N	%	*p*
**Toothbrushing**							
< 2 times/day	11	28.9	9	23.7	18	47.4	0.3
≥ 2 times/day	83	22.1	133	35.4	160	42.6	
Total	94	22.7	142	34.3	178	43.0	
**Daily dental floss**							
No	87	21.9	135	34.0	175	44.1	0.3
Yes	8	36.4	8	36.4	6	27.3	
Total	95	22.7	143	34.1	181	43.2	
**Dental appointment (last 12 months)**							
No	38	27.3	39	28.1	62	44.6	0.1
Yes	57	20.5	102	36.7	119	42.8	
Total	95	22.8	141	33.8	181	43.4	
**Fear of dental appointment**							
No	70	24.1	105	36.2	115	39.7	0.06
Yes	8	16.3	14	28.6	27	55.1	
Total	78	23.0	119	35.1	142	41.9	
**Experience of dental pain**							
No	72	25.4	99	34.9	113	39.8	0.02
Yes	23	16.8	45	32.8	69	50.4	
Total	95	22.6	144	34.2	182	43.2	
**Fissure sealants**							
No	17	13.5	37	29.4	72	57.1	0.001
Yes	55	30.6	69	38.3	56	31.1	
Total	72	23.5	106	34.6	128	41.9	

When questioning adolescents if they knew what a fissure sealant was, only 28.9% referred knowing what fissure sealants were and only 15.5% knew if their dentist had applied any on them in a past dental appointment.

When analyzing the prevalence of fissure sealants, we verified that 58.8% of adolescents had at least one fissure sealant applied. As shown in Table 4, from our total sample, we observed 830 teeth sealed, in which 63.3% were completely intact, 11.3% infiltrated but still without dental caries, 1.6% infiltrated but with the presence of dental caries, 23.5% with a partial fissure sealant but without dental caries and only 0.3% with a partial fissure sealant with dental caries present on the tooth surface.

**Table 4:** Assessment of the integrity of fissure sealants.

	N	%
Completely intact fissure sealant	526	63.3
Infiltrated fissure sealant without dental caries	94	11.3
Infiltrated fissure sealant with dental caries	13	1.6
Partial fissure sealant, without dental caries	195	23.5
Partial fissure sealant, with dental caries	3	0.3
Total	830	100.0

Through the analysis of the distribution of fissure sealants in different teeth groups we could verify that from the 830 identified fissure sealants, 20.0% were applied on the upper molars, 27.7% on the upper premolars, 27.6% on the lower molars and 24.7% on the lower premolars. These results lead us to conclude that there is an equal distribution of fissure sealants among teeth (Table 5).

**Table 5:** Assessment of the distribution of fissure sealants, by teeth groups.

	N	%
1^st^ and 2^nd^ upper molars	166	20.0
1^st^ and 2^nd^ upper premolars	230	27.7
1^st^ and 2^nd^ lower molars	229	27.6
1^st^ and 2^nd^ lower premolars	205	24.7
Total	830	100.0

When associating the presence of fissure sealants with sociodemographic variables, experience of dental pain and presence of dental caries, we can verify that the prevalence of fissure sealants was higher among adolescents whose parents had a higher level of education (>9^th^ grade, OR = 1.56 CI95% = 1.05–2.54), females (female, OR = 1.86 CI95% = 1.19–2.98), older adolescents (>15 years, OR = 1.44 CI95% = 0.82–2.53) and adolescents living in an urban area (urban, OR = 1.44 CI95% = 0.84–2.49). The prevalence of fissure sealants was lower among adolescents whose father was unemployed when the data collection took place (unemployed, OR = 0.94 CI95% = 0.43–2.09), that had experienced dental pain (yes, OR = 0.35 CI95% = 0.19–0.65) and with dental caries (yes, OR = 0.35 CI95% = 0.19–0.65). Significant statistical differences were found when analyzing the presence of fissure sealants related with parents´educational level, gender, experience of dental pain and presence of dental caries.

After adjustment by non-conditional logistic regression for sociodemographic variables, oral health behaviors (toothbrushing, daily use of dental floss and dental appointments) and experience of dental pain, the findings of the present study demonstrate that dental caries is associated with fissure sealant application (OR = 0.09 95%CI = 0.03–0.3).

## Discussion

In 2008, the General Health Directory of Portugal presented the last nationwide study of the prevalence of oral diseases. In that study, the DMFT index among adolescents aged 12 and 13 years was 1.48 and 3.04 respectively. The prevalence of dental caries increased with age and was also higher among the poorer socioeconomic classes.[7]

In this study we obtained a moderate level of prevalence of dental caries, with a higher number of filled teeth in comparison with the decayed and missing component of the DMFT index applied. This situation indicates that most of the adolescents have had, during their lives, dental appointments for dental caries treatment. These results demonstrate the improvement in oral healthcare registered in Portugal in the last years in which children and adolescents have easier access to dental appointments. However, the oral health indicators show the need of continuous improvement mainly towards primary prevention, specifically better oral hygiene habits.[6,7]

This can be obtained if oral health education initiatives are introduced in school programs, so that students and their families will be able to understand the importance of adequate oral hygiene habits, a balanced diet with less sugary food intake, beverage consumption and regular dental appointments. Another study developed by Barata *et al*. (2013) in another city of the central region of Portugal, obtained a DMFT index of 4.05 in adolescents with ages between 12 and 19. These results indicate the need of improvement in oral health behaviours among adolescents of this specific region of the country.[18] In Spain, a survey developed by Almerich Silla *et al*. (2006) indicates a caries prevalence of 55.9% among 15–16 year olds, but a DMFT index of 1.84. In this case, we can verify a higher caries prevalence, but a lower DMFT index when compared with the results obtained in this study.[19]

The prevalence of dental caries was highly associated with sociodemographic factors such as parents´ educational level. This association has been identified in several studies in which a lower socioeconomic status is not only associated with a higher risk of oral disease development, but also with worse oral health behaviours.[20–22]

Dental pain was also highly associated with higher levels of DMFT, which is also identified in various studies, and demonstrates that many adolescents have a dental appointment only in an emergency situation and maintain untreated dental caries.[23]

This study also showed that females had a higher DMFT than males, but a lower prevalence of dental caries at the moment of data collection. This demonstrates the higher care that female adolescents have on their oral health when compared with males. The higher rate of oral health care among females can also be justified by the existence of a higher prevalence of fissure sealant application among the females in comparison with males.[24,25] The fear of dental appointment was associated with lower application of fissure sealants, which can be explained by the lower frequency of routine check-up dental appointments. Studies show that adolescents and children that do not regularly visit a dentist do have a higher risk of dental caries development, failing, thus, the application of primary preventive measures that could prevent the appearance and development of dental caries and other oral diseases.[26,27]

The application of fissure sealants is associated with the higher risk of oral disease development and higher levels of DMFT. This can be explained by the fact that adolescents do not have proper access to adequate primary prevention methods. The correlation between dental caries and fissure sealants may be explained by the fact that adolescents with more dental caries and less fissure sealants are those that do not have regular dental appointments and do not benefit from the Portuguese oral health program that also includes the application of fissure sealants in permanent molars and premolars as a primary prevention method.[28] A study developed by Oulis *et al*. (2011) revealed that only 8.0% of Greek adolescents had at least one sealed molar, and the presence of fissure sealants was associated with a lower risk of dental caries prevalence.[29]

The application of fissure sealants is also associated in this study with parents´ educational level which is the main variable that permits the establishment of a significant statistical association between the application of primary prevention methods and socioeconomic status. Therefore, economic variables appear to be one of the reasons for the absence of fissure sealants among adolescents.[16,30,31]

When analysing the application of fissure sealants, we verify that less than one third of the adolescents know what a fissure sealant is. Several adolescents refer not knowing what a fissure sealant is, but do have fissure sealants applied to their own teeth. This can be justified by the fact that some adolescents do not understand what is being applied to their teeth during a dental appointment. Moreover, dentists do not always explain the treatment that is being done and the importance of the application of fissure sealants accurately. This reveals a low knowledge level of the definition and importance of primary prevention among adolescents and their families.[32] It is important to refer that 37.7% of the observed fissure sealants were not completely intact and were at risk of developing dental caries. Studies conclude that an infiltrated or a partial fissure sealant, when not detected early, can permit the development of dental caries under the reminiscent fissure sealant.[33, 34] This may occur because the majority of adolescents do not visit a dentist at least twice a year in order to reassess applied fissure sealants.[35]

Studies conducted by Griffin *et al*. (2010) analyzed dental caries risk in teeth with partially or totally lost sealants when compared to those that have never been sealed. The authors concluded that sealed teeth (either with completely or partially lost sealants) showed no greater risk of developing dental caries, when compared to those which have never been sealed. These results were conflicting and suggest a heightened concern because partially lost sealants may retain food debris and increase the risk of dental caries development.[36]

When considering fissure sealants, the earlier the application, the more effective they are. Therefore, in children fissure sealants are recommended to be applied soon after tooth eruption, mainly at the level of the first permanent molars.[8,37] Studies have shown that fissure sealants applied both in clinics and in schools, are highly effective in preventing dental caries, reducing caries in pits and fissures up to 60% for 2 to 5 years after its implementation.[38] A reassessment of fissure sealants should be held annually, not exceeding 12 months between dental appointments, for children and adolescents at high risk of developing dental caries. However, it is appropriate to reassess and reapply the fissure sealant within 6 months, in the particular cases of patients with high risk of developing dental caries and with insufficient oral health behaviors.[39]

In this specific population, the application of fissure sealants is an important complement of primary prevention because in Portugal there is no water fluoridation and the application of fluoride gel and varnish is not a common primary prevention treatment applied in dental medicine practice nowadays. This situation is verified in both urban and rural areas. However, previous studies have proven the use of fluoride toothpaste during daily oral hygiene by children and adolescents.[6,7]

In order to increase the knowledge of oral health behaviors and the application of primary prevention methods, more studies should be developed in Portugal nationwide to increase sample size and to better understand the reality of the Portuguese population. It is important to understand the determinants and distribution of oral diseases, but it is also important to identify the population with worse oral health behaviors in order to aim oral health education programmes towards the most problematic communities.

The application of primary preventive methods, namely fissure sealant application, in the dental appointment, complemented with oral health education, will certainly decrease the financial impact of oral treatments in the population and will guarantee a decrease in the risk of oral disease development during adulthood.

## Conclusions

The moderate level of caries prevalence devised in this study reveals the need of improvement as far primary prevention interventions among Portuguese adolescents are concerned. Oral health has improved in the last years in Portugal, especially among children and adults. However, the establishment of a more targeted preventive program with better and more effective oral health education is essential, bearing in mind sociodemographic aspects, with a special focus on adolescents and families with a lower socioeconomic status. The application of fissure sealants should be complemented with oral health education, in order for children, adolescents and their families to assimilate adequate oral hygiene habits and understand the need of regular dental appointments for primary prevention and early diagnosis of oral diseases.
